# Investigating the prediction potential of cholesterol, high-density lipoprotein, and glucose index surpasses TyG, AIP, and METS-IR for type 2 diabetes: a longitudinal cohort study

**DOI:** 10.3389/fendo.2026.1665074

**Published:** 2026-02-05

**Authors:** Si-Yuan Chen, Xiang-Xiang Li, Feng Lu, Hao Shen

**Affiliations:** 1 School of Medicine, Yangzhou University, Yangzhou, Jiangsu, China; 2 Tongli Community Health Center, Suzhou, Jiangsu, China; 3 Department of Clinical Laboratory, Suzhou Ninth Hospital Affiliated to Soochow University, Suzhou, Jiangsu, China; 4 Department of Pathogenic Biology and Immunology, School of Medicine, Yangzhou University, Yangzhou, Jiangsu, China; 5 Department of Laboratory Medicine, Wujiang Branch of Suzhou Central Blood Station, Suzhou, Jiangsu, China; 6 Department of Science and Technology, Suzhou Ninth Hospital Affiliated to Soochow University, Suzhou, Jiangsu, China

**Keywords:** atherogenic index of plasma, cholesterol, high-density lipoprotein, and glucose index, longitudinal cohort study, metabolic score for insulin resistance, triglyceride-glucose, type 2 diabetes mellitus

## Abstract

**Objective:**

To compare the predictive performance of the novel Cholesterol, High-Density Lipoprotein, and Glucose (CHG) index for the incident type 2 diabetes mellitus (T2DM) versus the established indices—triglyceride-glucose (TyG), atherogenic index of plasma (AIP), and metabolic score for insulin resistance (METS-IR).

**Methods:**

A longitudinal cohort study was performed on 15, 453 participants from the NAGALA cohort (2004–2015) without baseline T2DM. The CHG index was calculated from total cholesterol (TC), high-density lipoprotein cholesterol (HDL-C), and fasting blood glucose (FBG). Several additional indices were established, including the TyG, AIP, and METS-IR. Cox regression, Kaplan-Meier analysis, restricted cubic spline (RCS) analysis, and time-dependent receiver operating characteristic (ROC) curves were performed to determine the predictive performance, after adjusting for various confounders (e.g., obesity, liver function). Robustness was assessed through sensitivity analyses.

**Results:**

During a 6.13-year follow-up, 373 T2DM cases were recorded. In the fully adjusted models, we found that the CHG index had the highest hazard ratio (HR): 6.18 per unit (95% CI: 3.38–11.27), outperforming the TyG index (HR: 1.39), AIP (HR: 1.80), and METS-IR (HR: 1.07). Quartile analysis revealed a dose-response relationship for CHG (Q4 HR: 4.36 vs. Q1, *P* < 0.001). Further, the RCS analysis revealed a linear association between CHG and T2DM risk (*P* for overall trend < 0.001; *P* for nonlinearity = 0.712). Moreover, the CHG index achieved a satisfactory discriminative accuracy (based on the area under the curve [AUC]: 0.783 vs. TyG: 0.751, AIP: 0.745, METS-IR: 0.780) and its predictive power was consisted over the 12 years (peak AUC: 0.798 at 7 years). Sensitivity analyses for various subgroups (e.g., non-obese, non-fatty liver) confirmed that the obtained results were robust.

**Conclusions:**

The CHG index that integrates cholesterol, HDL-C, and glucose metabolism, showed superior predictive performance for the incidence of T2DM. Suggesting that it effectively captures the multifactorial pathophysiology of the disease. It is based on routine biomarkers making it easily applicable in risk stratification, offering a cost-effective tool for early intervention and precise prevention in clinical and public health settings.

## 1 Introduction

Globally, Type 2 diabetes mellitus (T2DM) presents a significant healthcare burden, with its prevalence escalating due to rising obesity rates, widespread sedentary behaviors, and the demographic shift toward older populations (1). The International Diabetes Federation estimated that nearly 536.6 million adults aged 20-79 (10.5% prevalence) had diabetes in 2021, a figure projected to reach 783.2 million (12.2%) by 2045 (2). Therefore, it is imperative to develop early identification tools for high-risk individuals and adopt preventive strategies. However, conventional biomarkers such as fasting plasma glucose (FPG) and hemoglobin A1c (HbA1c), have poor sensitivity in detecting early metabolic dysregulation and do not capture the multifaceted biological processes driving disease progression. Evidence indicates that initiating lifestyle modifications early can significantly mitigate or delay T2DM onset among high-risk individuals. Furthermore, early-stage screening alongside risk stratification supports these preventive measures (3). Thus, establishing simple and integrated screening tools will improve the identification of at-risk individuals to improve clinical decisions and public health policies, which is essential to reducing diabetes incidence and improving population health outcomes.

Insulin resistance (IR), which is a hallmark of T2DM characterized by impaired insulin signaling in peripheral tissues and dysregulated hepatic glucose production, is considered the key early risk prediction. This metabolic dysfunction not only precedes hyperglycemia but is also accompanied with lipid abnormalities, chronic inflammation, and oxidative stress, creating a complex network of interrelated mechanisms driving disease progression (4). Despite being the gold standard for quantifying IR, the hyperinsulinemic-euglycemic clamp (HEC) is constrained by its invasive procedure, high cost, and operational complexity, making it impractical for large-scale population screening (5). Similarly, indirect methods, such as the homeostasis model assessment of Insulin resistance (HOMA-IR) and quantitative insulin sensitivity check index (QUICKI) are limited by factors including lack of standardization and variability in insulin assays, which calls for the development of simpler, cost-effective alternative markers based on routinely measured biomarkers (6).

To address this gap, composite indices leveraging routine metabolic parameters have been formulated. The triglyceride-glucose (TyG) index, combining fasting triglycerides (TG) and glucose, can help to estimate peripheral IR but does not overlook cholesterol metabolism (7). The atherogenic index of plasma (AIP), derived from the TG-to-high density lipoprotein cholesterol (HDL-C) ratio, reflects atherogenic dyslipidemia, however, it does not consider direct glycemic measures (8). The metabolic score for insulin resistance (METS-IR) integrates body mass index (BMI), FPG, TG, and HDL-C which helps to link adiposity with lipid-glucose metabolism. Anthropometric measures like BMI and waist circumference are well-established, readily accessible indicators of adiposity and correlate strongly with insulin resistance and diabetes risk (9). However, its reliance on BMI can potentially mask the individual contributions of visceral fat (10). Although these indices have shown promising clinical utility, they often focus on isolated pathways—glucose-lipid interplay (TyG), atherogenic lipids (AIP), or adiposity-biased metrics (METS-IR), failing to reflect the synergistic interplay among various metabolic disruptions (e.g., cholesterol dysregulation, oxidative stress) which play important roles in the pathogenesis of T2DM. This shortcoming underscores the necessity for more comprehensive metrics that incorporate multiple metabolic parameters, thereby enhancing risk prognostication and early preventive approaches.

A recently developed composite biomarker, the Cholesterol, High-Density Lipoprotein, and Glucose (CHG) index, has been introduced as a promising diagnostic aid for T2DM, integrating key metabolic parameters into a single, potentially more indicative metric (11). A cross-sectional population study in Iran showed that this index performed better compared with the TyG index. However, in this study, the researchers enrolled individuals aged 35–65 years, did not compare emerging indices like the AIP and METS-IR, and lacked longitudinal data to establish causal relationships. To address these gaps, we leveraged longitudinal data from the NAGALA cohort to evaluate the CHG’s performance compared with the TyG, AIP, and METS-IR in predicting the incidence of T2DM (12). Participants from diverse age ranges were enrolled and several confounders such as liver function and obesity-related variables were adjusted for, which allowed us to validate CHG as a robust biomarker for T2DM risk prediction while investigating its ability to capture multifactorial metabolic dysregulation, including lipid-glucose interplay and systemic inflammation.

## 2 Methods

### 2.1 Study population

The NAGALA cohort, initiated in 1994 at Murakami Memorial Hospital (Japan), was designed to monitor the chronic disease risks using longitudinal health data. In the period 2004-2015, 20, 944 participants (12, 498 male/8, 446 female) were enrolled. After exclusion of cases with incomplete data (n=863), liver disease (n=416), excessive alcohol use (> 420 g/week male; > 280 g/week female, n=739), medication history (n=2, 321), diabetes/FPG ≥ 6.1 mmol/L (n=1, 131), non-response (n=10), or invalid HDL-C ratios (n=11), 15, 453 individuals (8, 419 male/7, 034 female) were retained for this study (enrollment flowchart: **Supplementary Figure S1**). Ethical approval and informed consent were obtained before patient enrolled. Approximately 60% of the patients received biennial diabetes/NAFLD screenings. The data used in this study were de-identified and were used to evaluate the novel CHG index for diabetes prediction, utilizing longitudinal follow-up to enhance risk assessment validity. This study utilized established methodologies previously described in the literature (12).

### 2.2 Collection of baseline data

Data collection was performed using standardized questionnaires, which were administered via trained personnel. Self-administered questionnaires captured key data across demographic characteristics, lifestyle factors (tobacco use, alcohol intake, exercise patterns), and medical histories. Smoking status was classified as never, former, or current smoker; alcohol intake was categorized by self-reported weekly consumption into non/small (< 40 g/week), light (40–140 g/week), moderate (140–280 g/week), and heavy (> 280 g/week); while regular exercise required ≥ 1 weekly physical activity session. Concurrently, trained personnel obtained anthropometric measurements—including height, body weight, waist circumference (WC), and blood pressure—following standardized protocols with participants wearing light indoor clothing without footwear. BMI values were obtained through the division of weight (kg) by height squared (m²). Venous blood samples were collected after ≥ 8-hour fasting and analyzed via automated biochemical analyzers to quantify: 1) lipid profile: total cholesterol (TC), TG, HDL-C, and low-density lipoprotein cholesterol (LDL-C) (calculated using Sampson’s equation (13)); 2) glucose parameters: FPG and HbA1c; 3) liver enzymes: aspartate aminotransferase (AST), alanine aminotransferase (ALT), and gamma-glutamyl transferase (GGT).

Fatty liver assessment via abdominal ultrasonography required dual-phase evaluation: technician-conducted scanning and gastroenterologist interpretation. Diagnostic determination utilized four sonographic features: ① liver brightness; ② hepatorenal echo contrast; ③ vascular blurring; ④ deep attenuation (12). T2DM was defined as HbA1c ≥ 6.5%, FPG ≥ 7.0 mmol/L, or self-reported diagnosis (14). However, the absence of oral glucose tolerance testing (OGTT) may have underestimated the incidence of T2DM. All procedures conformed to the ethical standards and the anonymization protocols were consistent with those reported in prior studies to ensure scientific rigor despite the diagnostic constraints.

### 2.3 Calculations and definitions

Metabolic indices were calculated using standardized formulas (all lipids in mg/dL):

Non-HDL-C = TC - HDL-C.

CHG index = Ln [TC × FBG/(2 × HDL-C)].

TyG index = Ln [TG × FBG/2].

METS-IR index = [Ln (2 × FPG + TG) × BMI]/Ln (HDL-C).

AIP index = log (TG/HDL-C).

### 2.4 Statistical analyses

All statistical analyses were performed using a quantitative framework integrating descriptive statistics and multi-model validation. We performed statistical analyses as follows. All the included variables violated normality assumptions upon analysis (Kolmogorov–Smirnov tests). Continuous variables with non-normal distribution used median (IQR) for data expression, analyzed by Mann-Whitney U (two-group comparisons) or Kruskal-Wallis tests (multi-group comparisons). Categorical data employed frequency (%) presentation with χ² tests for group contrasts. Kaplan-Meier curves with log-rank tests assessed time-to-T2DM onset.

The prognostic value of CHG for T2DM was assessed through progressively adjusted Cox models (unadjusted → fully adjusted). Covariate selection and adjustment were conducted based on the significant values obtained in the univariate analysis (*P* < 0.05) and a ≥ 10% change in hazard ratio (HR) upon covariate inclusion. FBG, HDL-C, and TC, which are constituent components of the CHG index, were excluded from the analysis to avoid theoretical conceptual overlap and potential over-adjustment of causal pathways between CHG and T2DM. Moreover, we included previously reported clinically significant covariates in the analysis. Then, we screened the selected covariates for collinearity (Variance Inflation Factor [VIF] < 5 for inclusion), and the VIF values of covariates in the final analysis are presented in **Supplementary Tables S1**, **S2** presents univariate cox analysis results with three adjusted models: Model 1 (Crude), Model 2 (Sex, Age, BMI), and Model 3 (Model 2 + Fatty liver, Exercise, Alcohol, Smoking, ALT, GGT, TG, LDL-C, HbA1c, SBP). Restricted cubic splines (RCS) with knots at 5th, 35th, 65th, and 95th percentiles of CHG explored linear and dose-response relationships. Sensitivity analyses (BMI ≥ 25, fatty liver exclusion) were conducted to ensure robustness of the results. Predictive capacity of CHG versus established indices (AIP, TyG, METS-IR) for T2DM was assessed by ROC-derived AUC quantification, with DeLong’s method determining statistical differences. Time-dependent ROC was conducted to assess the CHG’s predictive power for the occurrence of T2DM (1–12 years). All analyses used R 4.1.2, Zstats 1.0, and Free Statistics 2.0, with *P* < 0.05 (two-tailed) for significance.

## 3 Results

### 3.1 Baseline characteristics

Among the 15, 453 participants, 373 (2.4%) developed T2DM during a mean 6.13-year follow-up, and 15, 080 (97.6%) were non-T2DM. Participants with T2DM exhibited significantly higher median age (46.0 vs 42.0 years), BMI (24.7 vs 21.7 kg/m^2^), WC (85.0 vs 76.0 cm), body weight (69.0 vs 59.4 kg), liver enzymes (ALT 24.0 vs 17.0 U/L; GGT 24.0 vs 15.0 U/L), TG (107.0 vs 64.0 mg/dL), CHG (5.47 vs 5.09), and glycemic markers (HbA1c 5.6% vs 5.1%; FBG 103.0 vs 93.0 mg/dL) compared to non-T2DM participants (all *P* < 0.01). Lower HDL-C (43.7 vs 55.0 mg/dL) and male predominance (76.7% vs 53.9%) were detected in T2DM patients. The prevalence of fatty liver (59.8% vs 16.7%) was significantly (*P* < 0.01) different between the two groups, while exercise habits were not significantly different (*P* = 0.05) (**Supplementary Table S3**).

The analysis showed that all variables were significantly different among 15, 453 participants stratified by CHG quartiles (median [IQR]: Q1 = 4.72 [4.62–4.80], Q4 = 5.52 [5.43–5.65]) (*P* < 0.001). Higher quartiles exhibited progressively higher age (40.0 vs 45.0 years), BMI (19.85 vs 24.04 kg/m²), WC (69.5 vs 83.0 cm), weight (51.6 vs 68.6 kg), liver enzymes (ALT: 14.0 vs 23.0 U/L; GGT: 12.0 vs 21.0 U/L), lipids (TG:41.0 vs 119.0 mg/dL; LDL-C:98.36 vs 151.72 mg/dL), glycemic markers (HbA1c: 5.1% vs 5.2%; FBG: 88.0 vs 98.0 mg/dL), and blood pressure (SBP/DBP: 106.5/65.5 vs 120.5/76.0 mmHg), accompanied with the increase in CHG from 4.72 to 5.52 across quartiles. Concurrently, the concentration of HDL-C was reduced (71.0 vs 41.1 mg/dL). The male proportion increased substantially (23.04% vs 84.58%), accompanied by a marked rise in fatty liver prevalence (2.17% vs 43.56%). Furthermore, the rates of current smoking (11.0% vs 35.43%) and moderate to severe alcohol consumption (9.48% vs 14.44%) also increased, while exercise participation showed a modest decline (18.56% vs 15.66%). These dose-dependent trends, spanning a CHG range of 4.62 to 5.65, demonstrated strong associations between elevated CHG levels and adverse metabolic profiles, central obesity, and cardiometabolic risks (**Table 1**).

**Table 1:** Characteristics of the study population according to the quartiles of CHG.

Variables	Total (n = 15453)	Quartile 1 (n = 3863)	Quartile 2 (n = 3863)	Quartile 3 (n = 3863)	Quartile 4 (n = 3864)	*P*
Age, years	42.00 (37.00, 50.00)	40.00 (35.00, 46.00)	42.00 (36.00, 49.00)	44.00 (38.00, 52.00)	45.00 (39.00, 53.00)	<0.01
BMI, kg/m^2^	21.79 (19.89, 23.92)	19.85 (18.54, 21.41)	21.08 (19.60, 22.79)	22.44 (20.81, 24.29)	24.04 (22.31, 26.00)	<0.01
Waist circumference, cm	76.00 (70.00, 82.50)	69.50 (65.00, 74.10)	73.50 (68.50, 78.80)	78.50 (73.50, 83.50)	83.00 (78.47, 88.00)	<0.01
ALT, IU/L	17.00 (13.00, 23.00)	14.00 (11.00, 18.00)	15.00 (12.00, 19.00)	18.00 (14.00, 24.00)	23.00 (17.00, 32.00)	<0.01
AST, IU/L	17.00 (14.00, 21.00)	16.00 (13.00, 19.00)	17.00 (14.00, 20.00)	17.00 (14.00, 21.00)	19.00 (15.00, 23.00)	<0.01
Body Weight, kg	59.70 (51.80, 68.20)	51.60 (46.90, 57.40)	56.60 (50.40, 63.30)	62.60 (55.80, 69.40)	68.60 (61.90, 75.60)	<0.01
GGT, IU/L	15.00 (11.00, 22.00)	12.00 (10.00, 16.00)	13.00 (10.00, 18.00)	16.00 (12.00, 24.00)	21.00 (15.00, 32.00)	<0.01
HDL-C, mg/dL	54.60 (45.00, 66.00)	71.00 (63.00, 80.90)	59.50 (53.00, 66.70)	51.00 (45.45, 57.00)	41.10 (36.20, 46.10)	<0.01
TC, mg/dL	196.00 (174.00, 219.00)	179.00 (161.00, 199.00)	190.00 (171.00, 211.00)	200.00 (181.00, 221.00)	217.00 (197.00, 239.00)	<0.01
TG, mg/dL	65.00 (44.00, 99.00)	41.00 (30.50, 55.00)	55.00 (40.00, 73.00)	74.00 (56.00, 99.00)	119.00 (87.00, 165.00)	<0.01
Non-HDL-C, mg/dL	139.00 (116.30, 164.20)	107.00 (94.00, 122.00)	130.00 (115.80, 145.00)	148.90 (133.65, 164.90)	174.55 (157.38, 194.70)	<0.01
LDL-C, mg/dL	125.10 (104.83, 147.16)	98.36 (84.78, 113.26)	118.95 (104.78, 134.28)	134.29 (118.67, 150.42)	151.72 (133.69, 171.45)	<0.01
CHG	5.09 (4.86, 5.35)	4.72 (4.62, 4.80)	4.98 (4.92, 5.04)	5.22 (5.15, 5.28)	5.52 (5.43, 5.65)	<0.01
HbA1c, %	5.20 (5.00, 5.40)	5.10 (4.90, 5.30)	5.10 (4.90, 5.40)	5.20 (5.00, 5.40)	5.20 (5.00, 5.50)	<0.01
FBG, mg/dL	93.00 (88.00, 98.00)	88.00 (83.00, 92.00)	91.00 (87.00, 96.00)	94.00 (90.00, 99.00)	98.00 (93.00, 103.00)	<0.01
SBP, mmHg	113.00 (103.50, 124.00)	106.50 (99.00, 116.00)	111.00 (102.00, 120.50)	116.00 (107.00, 126.00)	120.50 (110.50, 130.50)	<0.01
DBP, mmHg	71.00 (64.00, 78.00)	65.50 (60.00, 72.50)	69.00 (63.00, 75.50)	72.50 (66.50, 79.50)	76.00 (69.50, 83.00)	<0.01
Sex, n(%)						<0.01
Women	7034 (45.52)	2973 (76.96)	2183 (56.51)	1282 (33.19)	596 (15.42)	
Men	8419 (54.48)	890 (23.04)	1680 (43.49)	2581 (66.81)	3268 (84.58)	
Fatty liver, n(%)						<0.01
No	12716 (82.29)	3779 (97.83)	3612 (93.50)	3144 (81.39)	2181 (56.44)	
Yes	2737 (17.71)	84 (2.17)	251 (6.50)	719 (18.61)	1683 (43.56)	
Habit of exercise, n(%)						<0.01
No	12747 (82.49)	3146 (81.44)	3139 (81.26)	3203 (82.91)	3259 (84.34)	
Yes	2706 (17.51)	717 (18.56)	724 (18.74)	660 (17.09)	605 (15.66)	
Alcohol consumption, n(%)						<0.01
Never	11802 (76.37)	3132 (81.08)	3004 (77.76)	2826 (73.16)	2840 (73.50)	
Light	1754 (11.35)	365 (9.45)	420 (10.87)	503 (13.02)	466 (12.06)	
Moderate	1357 (8.78)	280 (7.25)	315 (8.15)	374 (9.68)	388 (10.04)	
Severe	540 (3.49)	86 (2.23)	124 (3.21)	160 (4.14)	170 (4.40)	
Smoking status, n(%)						<0.01
Never	9027 (58.42)	2942 (76.16)	2565 (66.40)	1991 (51.54)	1529 (39.57)	
Past	2949 (19.08)	496 (12.84)	630 (16.31)	857 (22.18)	966 (25.00)	
Current	3477 (22.50)	425 (11.00)	668 (17.29)	1015 (26.27)	1369 (35.43)	

Continuous variables are presented as medians (interquartile range), while categorical variables are expressed as percentages (%).

BMI body mass index, WC Waist circumference, ALT alanine aminotransferase, AST aspartate aminotransferase, GGT gamma-glutamyl transferase, HDL-C high-density lipoprotein cholesterol, TC total cholesterol, TG triglyceride, LDL-C low-density lipid cholesterol, HbA1c hemoglobin A1c, FPG fasting plasma glucose, SBP systolic blood pressure, DBP diastolic blood pressure.

### 3.2 Associations of CHG, TyG, AIP, and METS-IR in various models

**Supplementary Table S2** summarizes the univariate Cox analyses for the CHG levels and T2DM risk. Notably, male sex (HR = 2.52), fatty liver (HR = 7.02), severe alcohol use (HR = 2.24), past/current smoking (HR = 1.65/2.58), age (HR = 1.06/year), BMI (HR = 1.24), elevated ALT, GGT, TG, LDL-C, CHG (HR = 19.14), HbA1c (HR = 54.27), and SBP (HR = 1.03) significantly increased T2DM risk (all *P* < 0.01). Exercise showed borderline protection (HR = 0.76, *P* = 0.06), while light/moderate alcohol consumption did not have a significant effect. CHG and HbA1c demonstrated exceptionally strong associations with T2DM development.

Baseline CHG values were assessed as predictors of incident T2DM using Cox proportional hazards modeling with multivariable adjustment (**Table 2**). Three models with incremental adjustments were applied to test the relationship. When CHG was analyzed as a continuous variable, a strong positive correlation was observed with the T2DM risk in all models. After full adjustment for sex, fatty liver, exercise habits, alcohol consumption, smoking status, age, BMI, ALT, GGT, TG, LDL-C, HbA1c, and SBP in Model 3, the HR remained statistically significant (HR: 6.18, 95% CI: 3.38–11.27, *P* < 0.001).

**Table 2:** The outcomes of multivariate analyses between baseline CHG levels and incident of T2DM.

Variables	Model 1			Model 2			Model 3	
	HR (95%CI)	*P*		HR (95%CI)	*P*		HR (95%CI)	*P*
CHG	19.14 (14.03~26.12)	<0.001		8.44 (5.78~12.32)	<0.001		6.18 (3.38 ~ 11.27)	<0.001
CHG quantile								
1	1.00 (Reference)			1.00 (Reference)			1.00 (Reference)	
2	3.13 (1.60 ~ 6.11)	<0.001		2.30 (1.17 ~ 4.50)	0.015		2.35 (1.19 ~ 4.64)	0.013
3	6.04 (3.21 ~ 11.37)	<0.001		3.16 (1.65 ~ 6.04)	<0.001		2.70 (1.38 ~ 5.29)	0.004
4	18.86 (10.31 ~ 34.50)	<0.001		7.11 (3.75 ~ 13.49)	<0.001		4.36 (2.15 ~ 8.83)	<0.001

HR, Hazard Ratio; CI, Confidence Interval.

Model1, Crude.

Model2, Adjust, Sex, Age, BMI.

Model3, Adjust, Sex, Fatty liver, Habit of exercise, Alcohol consumption, Smoking status, Age, BMI, ALT, GGT, TG, LDL-C, HbA1c, SBP.

**Figure 1:**
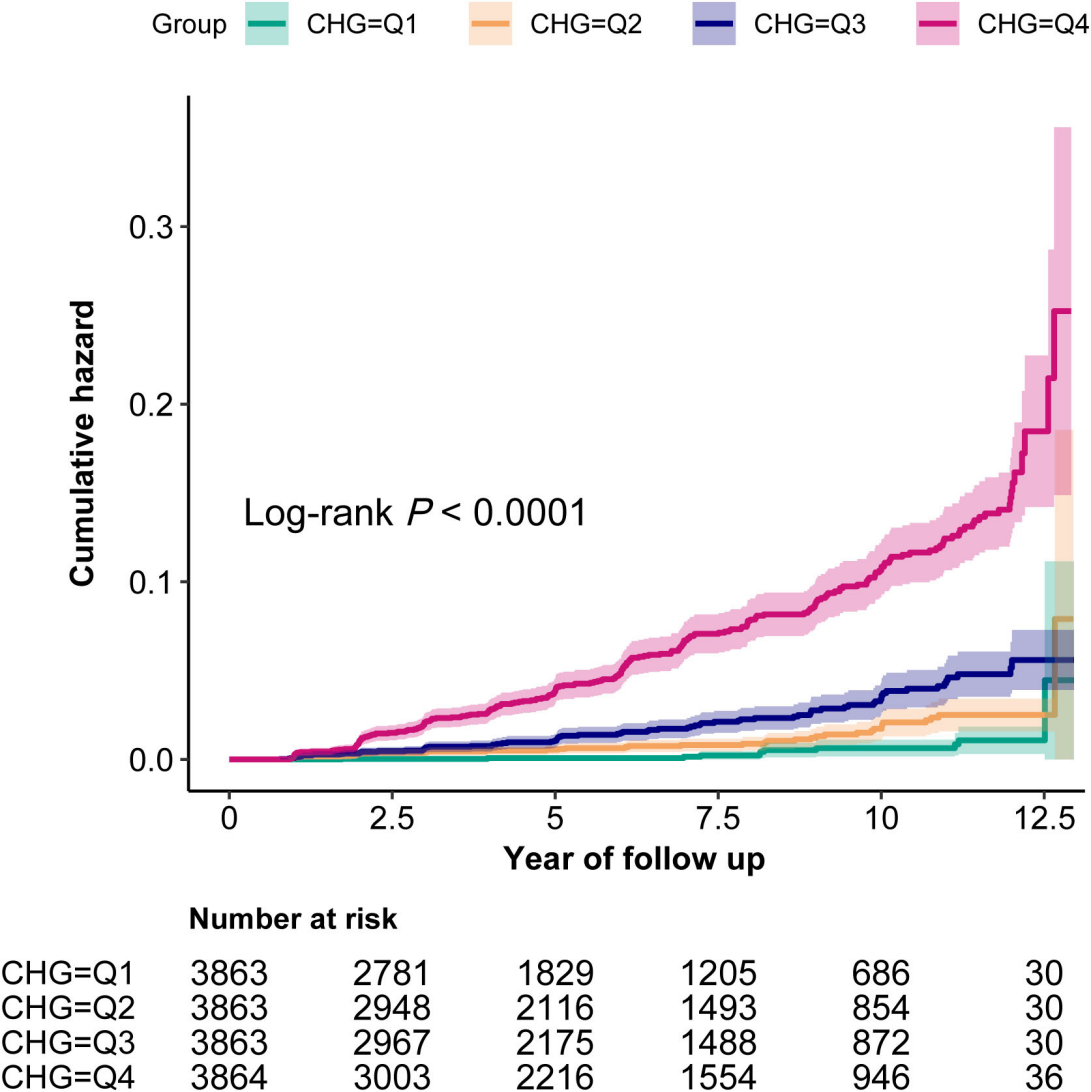
Kaplan-Meier analysis of cumulative T2DM incidence across CHG index quartiles. The survival curves demonstrate hierarchical cumulative hazards over 12.5 years of follow-up (log-rank *P* < 0.0001), revealing a dose-response relationship between CHG quartiles (Q1-Q4) and incident T2DM risk. Higher CHG quartiles (Q4) exhibited substantially elevated cumulative risk compared to the reference (Q1).

A dose-response relationship was observed when CHG was categorized into quantiles. Higher CHG quantiles, compared to the lowest quantile (reference), were associated with progressively increased risks of T2DM, even after full adjustment in Model 3 (Quantile 2: HR = 2.35, 95% CI: 1.19–4.64, *P* = 0.013; Quantile 3: HR = 2.70, 95% CI: 1.38–5.29, *P* = 0.004; Quantile 4: HR = 4.36, 95% CI: 2.15–8.83, *P* < 0.001). Kaplan-Meier curves revealed hierarchical T2DM incidence across groups (Q4>Q3>Q2>Q1, log-rank *P* < 0.0001, **Figure 1**).

### 3.3 Cox regression models for assessing the relationship between TyG index, AIP index, METS-IR index, and T2DM

Multivariable Cox regression analyses for four IR indices (CHG, TyG, AIP, METS-IR) indicated that the CHG had superior predictive potential for the incidence of T2DM (**Supplementary Tables S4-S6**). In fully adjusted models (Model 3), CHG demonstrated the highest HR (HR = 6.18, 95% CI: 3.38–11.27 per unit), significantly exceeding TyG (HR = 1.39), AIP (HR = 1.80), and METS-IR (HR = 1.07). We further analyzed multi-model COX regressions for TyG, AIP, and METS-IR and T2DM (the included covariates were screened for VIF). Quartile analysis demonstrated the discriminative power of CHG: the fourth quartile exhibited 4.36 times higher T2DM risk (95% CI: 2.15–8.83, *P* < 0.001), while other indices either showed non-significant risks (AIP Q4 HR = 1.20, *P* = 0.479; TyG Q4 HR = 1.20, *P* = 0.479) or attenuated risks (METS-IR Q4 HR = 3.69). There was modest attenuation from crude (HR = 19.14) to adjusted models, which underscored the CHG’s independence from metabolic confounders, further supporting its clinical relevance as the strongest biomarker for T2DM risk stratification.

### 3.4 Analysis of relationships between CHG and T2DM risk using RCS models

**Figure 2:**
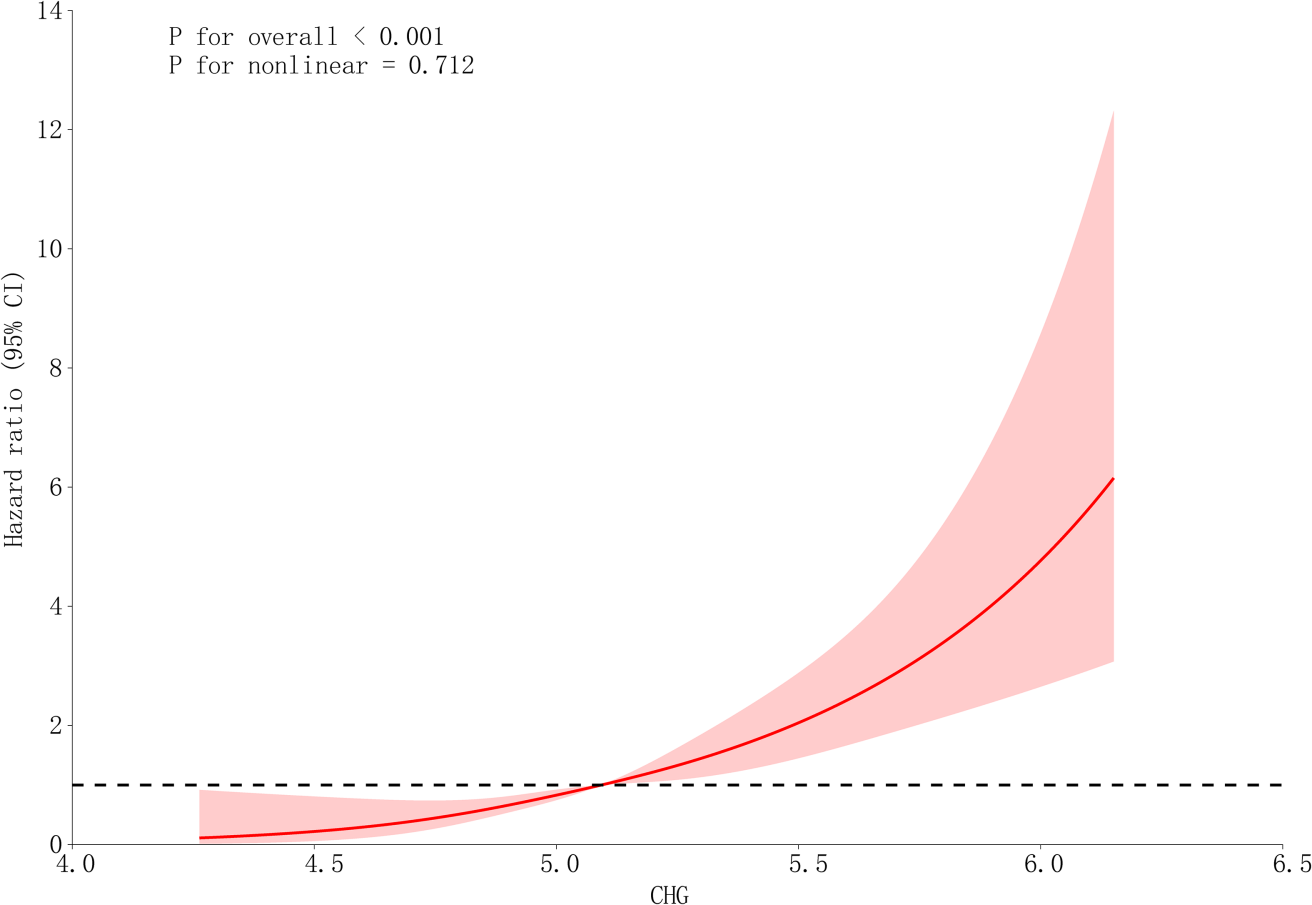
Dose-response relationship between CHG index and T2DM risk using restricted cubic spline (RCS) analysis. Multivariable-adjusted RCS curves show a linear association between CHG levels and T2DM risk (*P* for overall trend < 0.001; *P* for nonlinearity = 0.712). Hazard ratios increased progressively with CHG elevation, sharpening above CHG = 5.5. The analysis was adjusted for sex, fatty liver, habit of exercise, alcohol consumption, smoking status, age, BMI, ALT, GGT, TG, LDL-C, HbA1c, SBP.

A dose-dependent T2DM risk continuum was observed per unit CHG increase via adjusted RCS analyses (*P* for overall trend < 0.001; *P* for nonlinearity = 0.712) (**Figure 2**). The HR increased progressively with higher CHG levels, and this increase was particularly pronounced above CHG 5.5, indicating a linear association without significant nonlinear effects.

### 3.5 Sensitive analyses

In sensitivity analyses, we examining CHG’s association with incident T2DM, consistent positive trends were observed across all models. After excluding individuals with baseline BMI ≥ 25 kg/m² (Model 1), fatty liver (Model 2), or both (Model 3), higher CHG levels (quantiles 2–4 vs. reference) remained significantly associated with increased T2DM risk (HR: 2.53–7.50, all *P* < 0.05), see **Table 3**. These results demonstrated robust CHG-T2DM associations after application of exclusion criteria and covariate adjustments, indicating its independence from obesity- or fatty liver-related confounding.

**Table 3:** Association of CHG with the incidence of T2DM in sensitivity analyses of different clinical parameters.

Variables	Model 1 (n=12932)			Model 2 (n=12716)			Model 3 (n=11598)		
	HR (95% CI)	*P*		HR (95% CI)	*P*		HR (95% CI)	*P*	
RC	7.26 (3.23 ~ 16.29)	<0.001		8.98 (3.59 ~ 22.46)	<0.001		9.41 (3.41 ~ 25.97)	<0.001	
RC quantile									
1	1.00 (Reference)			1.00 (Reference)			1.00 (Reference)		
2	2.53 (1.12 ~ 5.70)	0.025		2.65 (1.12 ~ 6.26)	0.026		3.64 (1.35 ~ 9.78)	0.010	
3	3.00 (1.33 ~ 6.76)	0.008		2.74 (1.14 ~ 6.56)	0.024		2.94 (1.06 ~ 8.15)	0.039	
4	6.00 (2.54 ~ 14.13)	<0.001		6.32 (2.51 ~ 15.92)	<0.001		7.50 (2.61 ~ 21.57)	<0.001	

HR, Hazard Ratio; CI, Confidence Interval.

Model 1 presents a sensitivity analysis that excludes individuals with early-pregnancy BMI of ≥ 25 kg/m², adjusting for Sex, Fatty liver, Habit of exercise, Alcohol consumption, Smoking status, Age, BMI, ALT, GGT, TG, LDL-C, HbA1c, SBP.

Model 2 presents a sensitivity analysis that excludes individuals with Fatty liver, adjusting for Sex, Habit of exercise, Alcohol consumption, Smoking status, Age, BMI, ALT, GGT, TG, LDL-C, HbA1c, SBP.

Model 3 presents a sensitivity analysis using the exclusion criteria of the pooled model 1 + model 2, adjusting for Sex, Habit of exercise, Alcohol consumption, Smoking status, Age, BMI, ALT, GGT, TG, LDL-C, HbA1c, SBP.

### 3.6 Comparison of CHG with TyG, AIP, and METS-IR in predicting T2DM and time-dependent ROC analysis

**Figure 3:**
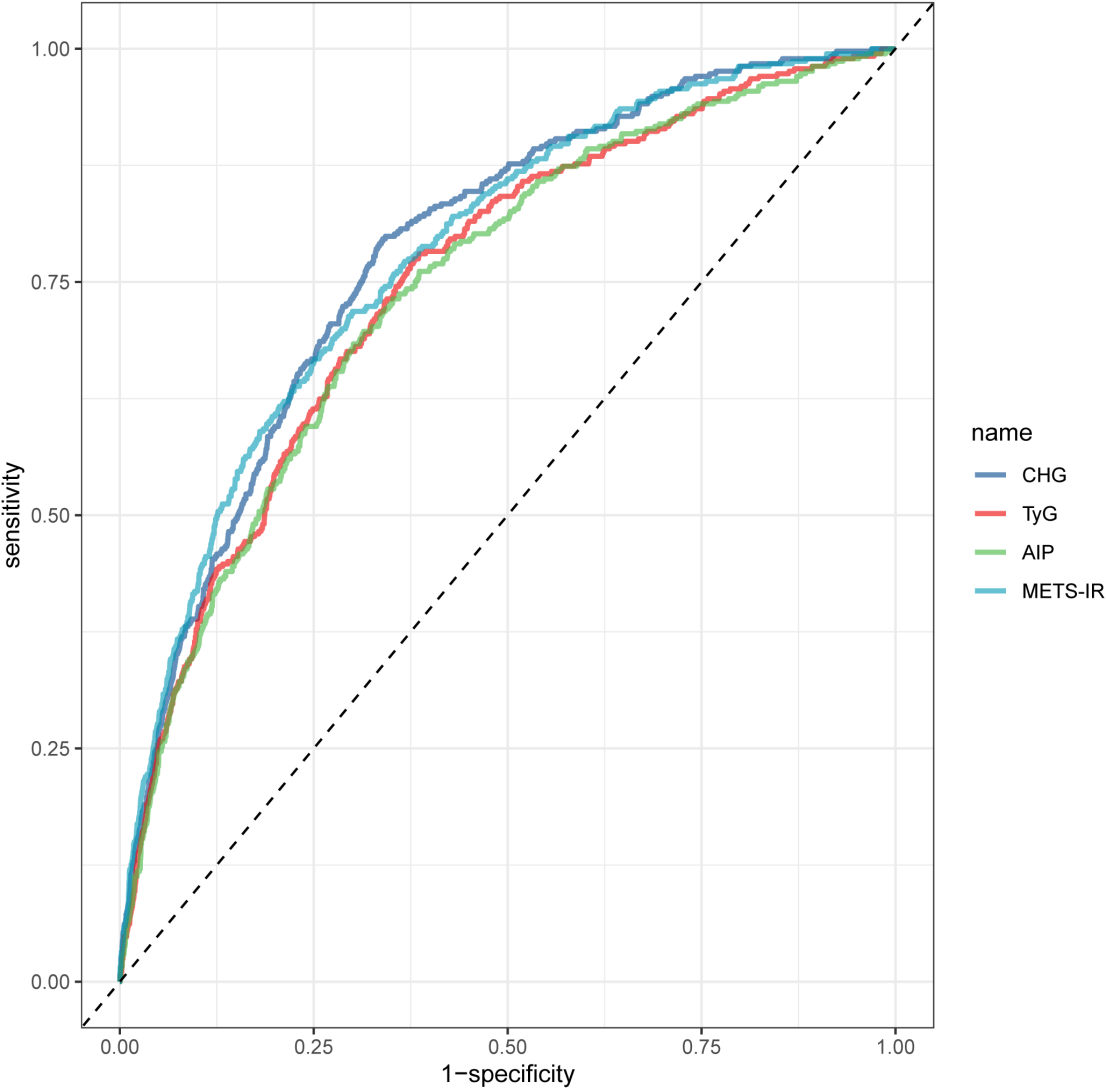
Receiver operating characteristic curves comparing predictive performance of CHG, TyG, AIP, and METS-IR indices. CHG achieved the highest discriminative accuracy (AUC = 0.783) for T2DM prediction, outperforming TyG (AUC = 0.751), AIP (AUC = 0.745), and METS-IR (AUC = 0.780). Optimal sensitivity (79.6%) and specificity (66.1%) were observed at the CHG cutoff value of 5.24.

Results of the ROC analysis indicated that CHG could predict GDM among the four indices (**Figure 3**, **Table 4**). Moreover, CHG achieved the highest AUC (0.783 vs. METS-IR 0.780, TyG 0.751, AIP 0.745), with a sensitivity of 79.6% and specificity of 66.1%, outperforming that of METS-IR (with a sensitivity of 71.9%) although it had marginally higher specificity (70.0%). In addition, the analysis demonstrated that the predictive performance of CHG for future T2DM risk, as measured by time-dependent AUC values, exhibited a dynamic pattern over the 12-year follow-up period (**Supplementary Figure S2**, **Supplementary Table S7**). The AUC increased from 0.737 at 1 year to a peak of 0.798 at 7 years, indicating improved discrimination over time. The AUC dipped modestly to 0.788 at 8 years but stayed relatively high. After year 9, predictive performance declined gradually, hitting 0.744 at year 11, though it saw a slight rebound to 0.765 by year 12. Notably, CHG exhibited a robust predictive accuracy (AUC >0.770) and stable confidence intervals (95% CI overlapping or above 0.730) from the 3rd to 8th year, which demonstrated consistent performance during this mid-term period. The highest AUC at 7 years (0.798, 95% CI 0.770–0.825) underscores CHG’s optimal performance in predicting T2DM risk during this interval. The comparative time-dependent AUC trajectories for TyG, AIP, and METS-IR are provided in **Supplementary Table S7**, offering a comprehensive view of each index’s discriminative ability over time.

**Table 4:** Receiver operating characteristic curve areas for each parameter in the identification of T2DM.

Parameter	AUC	95% CI low	95% CI up	Best threshold	Specificity	Sensitivity
CHG	0.783	0.760	0.805	5.242	0.661	0.796
TyG	0.751	0.726	0.775	8.196	0.620	0.775
AIP	0.745	0.720	0.770	0.235	0.686	0.697
METS-IR	0.780	0.756	0.803	33.634	0.700	0.719

AUC area under the curve, CI confidence interval, RC remnant cholesterol, HDL-C high-density lipoprotein cholesterol, TC total cholesterol, TG triglycerides, LDL-C low-density lipid cholesterol, METS-IR, metabolic score for insulin resistance, TyG index triglyceride-glucose index, AIP index atherogenic index of plasma.

## 4 Discussion

This longitudinal cohort study found that the novel CHG index showed better prediction performed on the incidence of T2DM relative to established indices such as the TyG, AIP, and METS-IR. Specifically, following adjustment for confounders such as obesity, liver function, and glycemic markers, CHG exhibited a 6.18-fold increased risk per unit elevation (95% CI: 3.38–11.27), significantly outperforming the TyG (HR = 1.39), AIP (HR = 1.80), and METS-IR (HR = 1.07). Sensitivity analyses indicated that these findings were robust, with CHG displaying a strong discriminative power during a 12-year follow-up period (time-dependent AUC = 0.74–0.80), with a sensitivity of 79.6% and specificity of 66.1% in comparison to other indices. These findings are consistent with prior evidence that integrated metabolic indices more effectively captures the multifactorial T2DM pathophysiology than single-domain biomarkers. Importantly, given that CHG is cost-effective and is based on routinely measured parameters, it provides an ideal tools for early screening and intervention in resource-limited settings, addressing the critical gap in global T2DM management.

Several studies have validated the utility of TyG, AIP, and METS-IR in the evaluating IR and metabolic dysregulation. The TyG index, which integrates lipid and glucose metabolism parameters, can accurately identify IR across diverse populations and is correlated with traditional markers like HOMA-IR in conditions such as gallstone disease (15, 16). The AIP is an indicator of atherogenic dyslipidemia and cardiovascular risk as it combines lipid profile abnormalities. METS-IR, which incorporates adiposity and metabolic parameters, has been shown to efficiently predict all-cause and cardiovascular mortality compared to other indices, particularly in non-elderly populations, and displays robust diagnostic utility in non-diabetic hypothyroidism (17, 18). In metabolic syndrome, these indices—especially TyG variants like TyG-WHtR—are significantly associated with hepatic steatosis and fibrosis risk (19). Together, these markers offer broad clinical utility, providing insights into IR, cardiovascular risk, and metabolic dysfunction in various populations without the need for direct insulin measurement.

Prior investigations have shown that TyG, AIP, and METS-IR are effective predictors of T2DM. For instance, meta-analyses and cohort studies have highlighted that TyG has high predictive value, with Qin et al. reporting a HR of 2.766 per TyG unit increase (20, 21). In addition, nonlinear associations were observed in some populations, where thresholds like TyG > 8.6 marked significant risk escalation (22). Similarly, AIP exhibited dose-dependent T2DM risks, with some meta-analyses indicating elevated AIP in T2DM patients (SMD = 1.78 vs. TG: 0.93) and longitudinal studies revealing J-shaped risk curves (threshold AIP ≥ -0.04, HR = 3.33) (23–25). The performance of METS-IR, that comprises lipids, glucose, and anthropometrics, varies across populations. Zhang et al. reported an 80% higher T2DM risk per standard deviation (SD) increase in METS-IR in rural Chinese adults (26), while Hou et al. obtained a 7% increase in the risk per unit in U.S. cohorts (27). However, Li et al. reported that METS-IR showed poorer performance compared with TyG in normoglycemic individuals (28), and Cheng et al. concluded that it had better value relative to the use of FBG alone, particularly in normoglycemic high-risk subgroups (29). These discrepancies reveal the context-dependent utility which varies depending on the population characteristics (age, ethnicity, metabolic profiles) and fluctuations in methodologies for measuring lipid-glucose interplay.

Compared to TyG, AIP, and METS-IR, the novel CHG index indicated better discrimination accuracy (AUC = 0.783 vs. TyG’s 0.751, AIP’s 0.745) and robust adjusted HR (6.18 vs. TyG’s 2.39, AIP’s 2.50), particularly in cohorts with hepatic comorbidities. Although the TyG primarily targets triglyceride-glucose dynamics and AIP on atherogenic lipids, CHG integrates TC, HDL-C, and FBG, integrating broad metabolic disturbances (e.g., cholesterol-driven IR), which are often overlooked by conventional indices. The CHG cohort, unlike previous cross-sectional studies (23, 27), benefits from longitudinal data with a 6.3-year follow-up and rigorous adjustment for confounders, including blood pressure and lipid profiles. Pan et al., for instance, identified TyG thresholds for risk stratification (22), CHG provides a continuous, holistic biomarker harmonizing lipid-glucose-cholesterol axes, making it widely applicable in multifactorial T2DM risk stratification in primary care settings. This integration improves early identification of high-risk individuals, even in normotensive or normoglycemic subgroups, suggesting that CHG is a clinically actionable tool for precision prevention.

Notably, the overall discriminative accuracy of CHG (AUC = 0.783) was closely matched by METS-IR (AUC = 0.780), underscoring that both are highly accurate predictive tools. It is important to note that while CHG and METS-IR showed comparable overall discriminative capacity, their clinical implications may differ. The significantly higher HR associated with CHG after full adjustment, coupled with its robust performance in sensitivity analyses excluding individuals with obesity (BMI ≥ 25 kg/m²), suggests that CHG may capture diabetes risk pathways that are less dependent on overall adiposity as reflected by BMI. This could make CHG particularly useful for identifying high-risk individuals within normal BMI ranges who might be overlooked by BMI-inclusive indices like METS-IR. Nevertheless, the high AUC of METS-IR reaffirms its value as a powerful composite metric, especially in settings where BMI is a routine and central component of metabolic assessment. It should be emphasized that the AUC values among the four parameters (CHG, TyG, AIP, and METS-IR) were notably similar, further supporting the utility of composite indices in T2DM risk stratification.

The clinical relevance of CHG extends beyond predicting T2DM onset, as evidenced by its association with diabetes-related complications. In a cross-sectional study of patients with long-standing T2DM, Çatak et al. (30) reported that the CHG index was significantly elevated in both diabetic retinopathy and nephropathy, and was the only index independently predictive of nephropathy—outperforming TyG, VAI, and LAP. This suggests that CHG may better capture the integrated metabolic stress underlying microvascular injury. Similarly, Mo et al. (31), in a large longitudinal cohort, found that the CHG index exhibited a linear dose-response relationship with cardiovascular disease risk, demonstrating predictive capability comparable to that of the TyG index. These findings collectively highlight the utility of CHG not only in predicting T2DM onset but also in stratifying risk for its microvascular and macrovascular sequelae. Given that a key goal of diabetes screening is the prevention of complications, the ability of CHG to reflect multiple pathophysiological pathways—glucose dysregulation, cholesterol metabolism, and HDL-related anti-inflammatory and antioxidant functions—supports its clinical relevance as a holistic biomarker for comprehensive risk assessment.

The high predictive power of CHG is based on its integration of multifaceted pathways that link IR, chronic inflammation, and oxidative stress. Dyslipidemia, a condition characterized by elevated TC and reduced HDL-C, exacerbates IR via inducing lipid metabolic dysfunction, macrophage-driven adipose inflammation, and proinflammatory cytokine release (e.g., TNF-α, IL-6), disrupting insulin signaling pathways (32, 33). Low HDL-C simultaneously exacerbates oxidative and endoplasmic reticulum (ER) stress, further impairing insulin sensitivity (34, 35). Research suggests that the reduced antioxidant capacity of HDL-C in T2DM contributes to increased oxidative stress, while lipid imbalances intensify ER stress, a key factor linked to IR (33, 36). Hyperglycemia tends to perpetuate this cycle by inducing oxidative stress, damaging pancreatic β-cell function, and worsening insulin secretion and action (37). Therefore, the CHG’s potential to capture these interlinked mechanisms, lipid dysregulation, inflammatory cascades, and oxidative damage, suggests that it is a robust predictor of T2DM progression.

Beyond its predictive superiority, the CHG index holds significant translational potential for real-world T2DM prevention strategies. Its derivation from routinely measured biomarkers (TC, HDL-C, FBG) enables seamless integration into existing clinical workflows without additional costs or specialized equipment—a critical advantage for resource-constrained settings. This accessibility could facilitate widespread implementation in primary care screenings, particularly for identifying high-risk individuals with normal BMI but adverse lipid-glucose profiles who are often overlooked by conventional adiposity-centric tools (e.g., METS-IR). Furthermore, CHG’s linear dose-response relationship supports straightforward risk stratification using clinically actionable thresholds (e.g., CHG > 5.24), empowering clinicians to initiate timely interventions such as lifestyle modification or HDL-C-targeted therapies.

This study has major advantages. First, it provides longitudinal evidence regarding the CHG’s temporal relationship with T2DM onset, which overcomes the inherent limitations of cross-sectional studies in establishing causality. Therefore, we utilized a robust analytical framework—integrating survival analysis, sensitivity testing, and comparative benchmarking—to validate the CHG’s diagnostic superiority over conventional indices (TyG, AIP, METS-IR) in both discriminative accuracy and clinical interpretability. Furthermore, comprehensive adjustment for various metabolic confounders (e.g., hepatic dysfunction, adiposity-related variables) and advanced statistical modeling were adopted to clarify the linear risk gradients, ensuring reproducibility across heterogeneous populations. Finally, the translational potential of CHG is enhanced by its operational simplicity, which leverages routinely measured biomarkers to facilitate scalable risk stratification in resource-limited settings. Moreover, its potential to inform early preventive interventions is consistent with the global need for precise public health interventions.

Nevertheless, interpretation of the results should be tempered by three key study limitations. Notably, as our cohort comprised Japanese adults, the generalizability of our findings to other ethnic populations (particularly those with different average BMI profiles and body composition, such as Latino or African groups) may be limited, and future studies are needed to validate the CHG index in these specific populations. Furthermore, the study’s methodological limitations include the use of HbA1c and FBG without OGTT. This approach likely leads to a systematic underestimation of T2DM incidence by failing to identify a non-negligible subgroup of individuals with isolated postprandial hyperglycemia, who are often characterized by early-phase insulin secretion defects and peripheral insulin resistance (38, 39). Consequently, the reported predictive performance of the CHG index, which incorporates FBG, may be most robust for identifying T2DM cases accompanied by fasting hyperglycemia and might not fully capture its utility in the broader spectrum of dysglycemia. Therefore, further studies are warranted to validate and potentially recalibrate its predictive power across the full range of glucose metabolic disorders. Moreover, the absence of gold-standard IR assessments, such as HEC, limits the ability to mechanistically validate CHG’s association with peripheral/hepatic insulin signaling pathways. Moreover, unmeasured confounders—such as dietary macronutrient patterns, gut microbiota dysbiosis, and epigenetic regulators—may introduce residual bias, while static CHG measurements do not capture dynamic risk trajectories associated with longitudinal metabolic fluctuations. To address these gaps, future research should prioritize: (1) conduct multicenter validation across global populations to establish ethnicity-specific risk thresholds; (2) integrate multi-omics profiling (metabolomics, proteomics) to explore the CHG-associated molecular networks; (3) perform longitudinal monitoring of CHG dynamics to refine time-dependent risk algorithms; and (4) develop AI-driven models combining CHG with radiomic features (hepatic fat quantification) and digital health metrics (continuous glucose monitoring) for precision prediction. Such investigations will solidify the CHG’s role in equitable, evidence-based diabetes prevention.

## 5 Conclusions

In this study, we found that the novel CHG index showed better performance in predicting the incidence of T2DM compared to traditional metabolic indices. The index integrates cholesterol homeostasis (TC, HDL-C) and FPG, and CHG effectively captures the multifactorial pathophysiology, including aspects, such as IR and lipid-inflammatory crosstalk. Clinically, CHG offers a cost-effective, practical tool that uses the measured parameters, enabling early identification of high-risk individuals—even those with normal BMI but subclinical metabolic disturbances. Its comprehensive approach facilitates the development of targeted interventions, including HDL-C modulation and cholesterol-lowering strategies, aimed at interrupting disease progression in prediabetic populations. CHG harmonizes the key metabolic pathways, addressing T2DM complexity unlike conventional single-domain biomarkers, thereby enhancing disease prevention.

## Data Availability

The datasets presented in this study can be found in online repositories. The names of the repository/repositories and accession number(s) can be found below: https://datadryad.org/stash/dataset/doi:10.5061%2Fdryad.8q0p192.
