# Supplementary material for: Investigating the prediction potential of cholesterol, high-density lipoprotein, and glucose index surpasses TyG, AIP, and METS-IR for type 2 diabetes: a longitudinal cohort study
Source: Front Endocrinol (Lausanne). 2026 Feb 5;17:1665074. doi: 10.3389/fendo.2026.1665074 (PMC12916360; doi:10.3389/fendo.2026.1665074)
Supplement: Supplementary file 1 [file Table1.docx]

***Supplementary Material***

**Supplementary Table 1** Collinearity test in multifactor cox regressions.

| Variables | VIF | colinearity |
| --- | --- | --- |
| CHG | 3.521 | 0 |
| Age | 1.225 | 0 |
| Sex | 1.849 | 0 |
| Fatty liver | 1.578 | 0 |
| Habit of exercise | 1.04 | 0 |
| Alcohol consumption | 1.406 | 0 |
| Smoking status | 1.675 | 0 |
| SBP | 1.405 | 0 |
| BMI | 1.801 | 0 |
| HbA1c | 1.157 | 0 |
| GGT | 1.406 | 0 |
| ALT | 1.444 | 0 |
| TG | 2.054 | 0 |
| LDL-C | 1.818 | 0 |

The variance inflation factor test was used to evaluate the collinearity problem respectively. VIF variance inflation factor. VIF of all parameters was less than 5, indicating that there was no serious collinearity problem.

**Supplementary Table 2** The outcomes of univariate cox analyses between CHG levels and the risk of T2DM.

| Variables | β | S.E | Z | *P* | HR (95%CI) |
| --- | --- | --- | --- | --- | --- |
| Sex |  |  |  |  |  |
| Women |  |  |  |  | 1.00 (Reference) |
| Men | 0.93 | 0.12 | 7.55 | **<0.01** | 2.52 (1.98 ~ 3.21) |
| Fatty liver |  |  |  |  |  |
| No |  |  |  |  | 1.00 (Reference) |
| Yes | 1.95 | 0.11 | 18.44 | **<0.01** | 7.02 (5.70 ~ 8.63) |
| Habit of exercise |  |  |  |  |  |
| No |  |  |  |  | 1.00 (Reference) |
| Yes | -0.28 | 0.15 | -1.85 | 0.06 | 0.76 (0.56 ~ 1.02) |
| Alcohol consumption |  |  |  |  |  |
| Never |  |  |  |  | 1.00 (Reference) |
| Light | -0.10 | 0.17 | -0.60 | 0.55 | 0.90 (0.65 ~ 1.26) |
| Moderate | 0.14 | 0.18 | 0.80 | 0.42 | 1.15 (0.82 ~ 1.62) |
| Severe | 0.81 | 0.19 | 4.19 | **<0.01** | 2.24 (1.54 ~ 3.27) |
| Smoking status |  |  |  |  |  |
| Never |  |  |  |  | 1.00 (Reference) |
| Past | 0.50 | 0.14 | 3.57 | **<0.01** | 1.65 (1.26 ~ 2.18) |
| Current | 0.95 | 0.12 | 8.16 | **<0.01** | 2.58 (2.06 ~ 3.24) |
| Age | 0.05 | 0.01 | 9.11 | **<0.01** | 1.06 (1.04 ~ 1.07) |
| BMI | 0.22 | 0.01 | 20.16 | **<0.01** | 1.24 (1.22 ~ 1.27) |
| ALT | 0.01 | 0.00 | 11.18 | **<0.01** | 1.01 (1.01 ~ 1.01) |
| GGT | 0.01 | 0.00 | 10.48 | **<0.01** | 1.01 (1.01 ~ 1.01) |
| TG | 0.01 | 0.00 | 17.12 | **<0.01** | 1.01 (1.01 ~ 1.01) |
| LDL-C | 0.01 | 0.00 | 8.30 | **<0.01** | 1.01 (1.01 ~ 1.01) |
| CHG | 2.95 | 0.16 | 18.61 | **<0.01** | 19.14 (14.03 ~ 26.12) |
| HbA1c | 3.99 | 0.16 | 24.62 | **<0.01** | 54.27 (39.49 ~ 74.59) |
| SBP | 0.03 | 0.00 | 11.02 | **<0.01** | 1.03 (1.03 ~ 1.04) |

HR: Hazard Ratio, CI: Confidence Interval.

BMI body mass index, WC Waist circumference, ALT alanine aminotransferase, AST aspartate aminotransferase, GGT gamma-glutamyl transferase, HDL-C high-density lipoprotein cholesterol, TC total cholesterol, TG triglyceride, LDL-C low-density lipid cholesterol, HbA1c hemoglobin A1c, SBP systolic blood pressure, DBP diastolic blood pressure.

**Supplementary Table 3** Clinical characteristics between T2DM and non-T2DM in participants.

| Variables | Total (n = 15453) | Non-T2DM (n = 15080) | T2DM (n = 373) | *P* |
| --- | --- | --- | --- | --- |
|  |  |  |  |  |
| Age, years | 42.00 (37.00, 50.00) | 42.00 (37.00, 50.00) | 46.00 (41.00, 53.00) | **<0.01** |
| BMI, kg/m^2^ | 21.79 (19.89, 23.92) | 21.74 (19.86, 23.83) | 24.66 (22.29, 27.25) | **<0.01** |
| Waist circumference, cm | 76.00 (70.00, 82.50) | 76.00 (69.80, 82.00) | 85.00 (77.50, 91.30) | **<0.01** |
| ALT, IU/L | 17.00 (13.00, 23.00) | 17.00 (13.00, 23.00) | 24.00 (18.00, 39.00) | **<0.01** |
| AST, IU/L | 17.00 (14.00, 21.00) | 17.00 (14.00, 21.00) | 20.00 (16.00, 26.00) | **<0.01** |
| Body Weight, kg | 59.70 (51.80, 68.20) | 59.40 (51.60, 67.90) | 69.00 (60.00, 78.00) | **<0.01** |
| GGT, IU/L | 15.00 (11.00, 22.00) | 15.00 (11.00, 22.00) | 24.00 (17.00, 36.00) | **<0.01** |
| HDL-C, mg/dL | 54.60 (45.00, 66.00) | 55.00 (45.40, 66.00) | 43.70 (37.30, 51.20) | **<0.01** |
| TC, mg/dL | 196.00 (174.00, 219.00) | 196.00 (174.00, 219.00) | 210.00 (186.00, 232.00) | **<0.01** |
| TG, mg/dL | 65.00 (44.00, 99.00) | 64.00 (43.00, 98.00) | 107.00 (76.00, 171.00) | **<0.01** |
| Non-HDL-C, mg/dL | 139.00 (116.30, 164.20) | 138.70 (116.00, 163.40) | 162.60 (137.90, 186.30) | **<0.01** |
| LDL-C, mg/dL | 125.10 (104.83, 147.16) | 124.76 (104.52, 146.67) | 139.74 (119.27, 162.46) | **<0.01** |
| CHG | 5.09 (4.86, 5.35) | 5.09 (4.86, 5.34) | 5.47 (5.27, 5.68) | **<0.01** |
| HbA1c, % | 5.20 (5.00, 5.40) | 5.15 (4.94, 5.40) | 5.60 (5.30, 5.80) | **<0.01** |
| FBG, mg/dL | 93.00 (88.00, 98.00) | 93.00 (88.00, 98.00) | 103.00 (97.00, 106.00) | **<0.01** |
| SBP, mmHg | 113.00 (103.50, 124.00) | 113.00 (103.50, 123.50) | 121.50 (112.00, 132.00) | **<0.01** |
| DBP, mmHg | 71.00 (64.00, 78.00) | 70.50 (64.00, 78.00) | 76.50 (70.00, 84.00) | **<0.01** |
| Sex, n(%) |  |  |  | **<0.01** |
| Women | 7034 (45.52) | 6947 (46.07) | 87 (23.32) |  |
| Men | 8419 (54.48) | 8133 (53.93) | 286 (76.68) |  |
| Fatty liver, n(%) |  |  |  | **<0.01** |
| No | 12716 (82.29) | 12566 (83.33) | 150 (40.21) |  |
| Yes | 2737 (17.71) | 2514 (16.67) | 223 (59.79) |  |
| Habit of exercise, n(%) |  |  |  | **0.05** |
| No | 12747 (82.49) | 12425 (82.39) | 322 (86.33) |  |
| Yes | 2706 (17.51) | 2655 (17.61) | 51 (13.67) |  |
| Alcohol consumption, n(%) |  |  |  | **<0.01** |
| Never | 11802 (76.37) | 11536 (76.50) | 266 (71.31) |  |
| Light | 1754 (11.35) | 1714 (11.37) | 40 (10.72) |  |
| Moderate | 1357 (8.78) | 1320 (8.75) | 37 (9.92) |  |
| Severe | 540 (3.49) | 510 (3.38) | 30 (8.04) |  |
| Smoking status, n(%) |  |  |  | **<.01** |
| Never | 9027 (58.42) | 8882 (58.90) | 145 (38.87) |  |
| Past | 2949 (19.08) | 2872 (19.05) | 77 (20.64) |  |
| Current | 3477 (22.50) | 3326 (22.06) | 151 (40.48) |  |

Continuous variables are presented as medians (interquartile range), while categorical variables are expressed as percentages (%).

BMI body mass index, WC Waist circumference, ALT alanine aminotransferase, AST aspartate aminotransferase, GGT gamma-glutamyl transferase, HDL-C high-density lipoprotein cholesterol, TC total cholesterol, TG triglyceride, LDL-C low-density lipid cholesterol, HbA1c hemoglobin A1c, FPG fasting plasma glucose, SBP systolic blood pressure, DBP diastolic blood pressure.

**Supplementary Table 4** The outcomes of multivariate analyses between baseline TyG levels and incident of T2DM.

| Variables | Model 1 | |  | Model 2 | |  | Model 3 | |
| --- | --- | --- | --- | --- | --- | --- | --- | --- |
|  | HR (95%CI) | *P* |  | HR (95%CI) | *P* |  | HR (95%CI) | *P* |
| TyG | 3.76 (3.22 ~ 4.38) | **<0.001** |  | 2.39 (1.99 ~ 2.88) | **<0.001** |  | 1.39 (1.11 ~ 1.73) | **0.003** |
| TyG quantile |  |  |  |  |  |  |  |  |
| 1 | 1.00 (Reference) |  |  | 1.00 (Reference) |  |  | 1.00 (Reference) |  |
| 2 | 1.34 (0.80 ~ 2.25) | 0.270 |  | 0.92 (0.55 ~ 1.56) | 0.763 |  | 0.76 (0.44 ~ 1.29) | 0.304 |
| 3 | 3.29 (2.10 ~ 5.17) | **<0.001** |  | 1.61 (1.01 ~ 2.57) | **0.048** |  | 1.05 (0.64 ~ 1.72) | 0.839 |
| 4 | 8.21 (5.39 ~ 12.51) | **<0.001** |  | 2.84 (1.80 ~ 4.50) | **<0.001** |  | 1.20 (0.72 ~ 1.99) | 0.479 |

HR: Hazard Ratio, CI: Confidence Interval.

Model1: Crude.

Model2: Adjust: Sex, Age, BMI.

Model3: Adjust: Sex, Fatty liver, Habit of exercise, Alcohol consumption, Smoking status, Age, BMI, ALT, GGT, HDL-C, LDL-C, HbA1c, SBP.

**Supplementary Table 5** The outcomes of multivariate analyses between baseline AIP levels and incident of T2DM.

| Variables | Model 1 | |  | Model 2 | |  | Model 3 | |
| --- | --- | --- | --- | --- | --- | --- | --- | --- |
|  | HR (95%CI) | *P* |  | HR (95%CI) | *P* |  | HR (95%CI) | *P* |
| AIP | 10.26 (7.71 ~ 13.65) | **<0.001** |  | 4.28 (3.01 ~ 6.08) | **<0.001** |  | 1.80 (1.23 ~ 2.61) | **<0.001** |
| AIP quantile |  |  |  |  |  |  |  |  |
| 1 | 1.00 (Reference) |  |  | 1.00 (Reference) |  |  | 1.00 (Reference) |  |
| 2 | 1.78 (1.07 ~ 2.95) | **0.026** |  | 1.23 (0.74 ~ 2.06) | 0.421 |  | 0.99 (0.59 ~ 1.66) | 0.972 |
| 3 | 3.04 (1.90 ~ 4.87) | **<0.001** |  | 1.51 (0.93 ~ 2.46) | 0.099 |  | 0.91 (0.55 ~ 1.50) | 0.719 |
| 4 | 7.99 (5.15 ~ 12.38) | **<0.001** |  | 2.68 (1.66 ~ 4.34) | **<0.001** |  | 1.20 (0.73 ~ 1.97) | 0.479 |

HR: Hazard Ratio, CI: Confidence Interval.

Model1: Crude.

Model2: Adjust: Sex, Age, BMI.

Model3: Adjust: Sex, Fatty liver, Habit of exercise, Alcohol consumption, Smoking status, Age, BMI, ALT, GGT, TC, HbA1c, FBG, SBP.

**Supplementary Table 6** The outcomes of multivariate analyses between baseline METS-IR levels and incident of T2DM.

| Variables | Model 1 | |  | Model 2 | |  | Model 3 | |
| --- | --- | --- | --- | --- | --- | --- | --- | --- |
|  | HR (95%CI) | *P* |  | HR (95%CI) | *P* |  | HR (95%CI) | *P* |
| METS-IR | 1.13 (1.12 ~ 1.15) | **<0.001** |  | 1.14 (1.13 ~ 1.15) | **<0.001** |  | 1.07 (1.05 ~ 1.09) | **<0.001** |
| METS-IR quantile |  |  |  |  |  |  |  |  |
| 1 | 1.00 (Reference) |  |  | 1.00 (Reference) |  |  | 1.00 (Reference) |  |
| 2 | 2.69 (1.46 ~ 4.95) | **0.001** |  | 2.42 (1.31 ~ 4.46) | **0.005** |  | 2.26 (1.22 ~ 4.18) | **0.009** |
| 3 | 4.99 (2.82 ~ 8.81) | **<0.001** |  | 4.06 (2.27 ~ 7.28) | **<0.001** |  | 2.31 (1.27 ~ 4.20) | **0.006** |
| 4 | 15.52 (9.05 ~ 26.60) | **<0.001** |  | 12.81 (7.31 ~ 22.44) | **<0.001** |  | 3.69 (2.02 ~ 6.74) | **<0.001** |

HR: Hazard Ratio, CI: Confidence Interval.

Model1: Crude.

Model2: Adjust: Sex, Age, BMI.

Model3: Adjust: Sex, Fatty liver, Habit of exercise, Alcohol consumption, Smoking status, Age, ALT, GGT, LDL-C, HbA1c, SBP.

**Supplementary Table 7** Areas under the time-dependent ROC curves for CHG, TyG, AIP, and METS-IR indices in predicting future T2DM risk over 12 years.

| Years | CHG | | TyG | | AIP | | METS-IR | |
| --- | --- | --- | --- | --- | --- | --- | --- | --- |
|  | AUC | 95% CI | AUC | 95% CI | AUC | 95% CI | AUC | 95% CI |
| 1 | 0.737 | 0.642–0.832 | 0.707 | 0.603–0.811 | 0.690 | 0.582–0.799 | 0.722 | 0.628–0.815 |
| 2 | 0.749 | 0.688–0.810 | 0.734 | 0.662–0.805 | 0.710 | 0.634–0.786 | 0.724 | 0.662–0.786 |
| 3 | 0.783 | 0.741–0.825 | 0.738 | 0.688–0.788 | 0.737 | 0.687–0.787 | 0.767 | 0.719–0.813 |
| 4 | 0.772 | 0.732–0.811 | 0.723 | 0.676–0.770 | 0.711 | 0.663–0.759 | 0.751 | 0.706–0.795 |
| 5 | 0.787 | 0.754–0.820 | 0.735 | 0.696–0.774 | 0.725 | 0.686–0.764 | 0.780 | 0.742–0.817 |
| 6 | 0.789 | 0.759–0.818 | 0.741 | 0.706–0.777 | 0.734 | 0.698–0.769 | 0.788 | 0.755–0.820 |
| 7 | 0.798 | 0.770–0.825 | 0.754 | 0.721–0.787 | 0.745 | 0.713–0.778 | 0.799 | 0.770–0.828 |
| 8 | 0.788 | 0.761–0.815 | 0.747 | 0.715–0.778 | 0.738 | 0.707–0.769 | 0.799 | 0.772–0.827 |
| 9 | 0.766 | 0.737–0.795 | 0.737 | 0.707–0.766 | 0.723 | 0.693–0.754 | 0.777 | 0.749–0.806 |
| 10 | 0.755 | 0.726–0.784 | 0.725 | 0.694–0.756 | 0.711 | 0.679–0.742 | 0.771 | 0.742–0.801 |
| 11 | 0.744 | 0.714–0.773 | 0.719 | 0.687–0.751 | 0.698 | 0.664–0.731 | 0.759 | 0.729–0.788 |
| 12 | 0.737 | 0.732–0.799 | 0.720 | 0.684–0.756 | 0.719 | 0.674–0.749 | 0.769 | 0.737–0.802 |

AUC area under the curve, CI confidence interval.


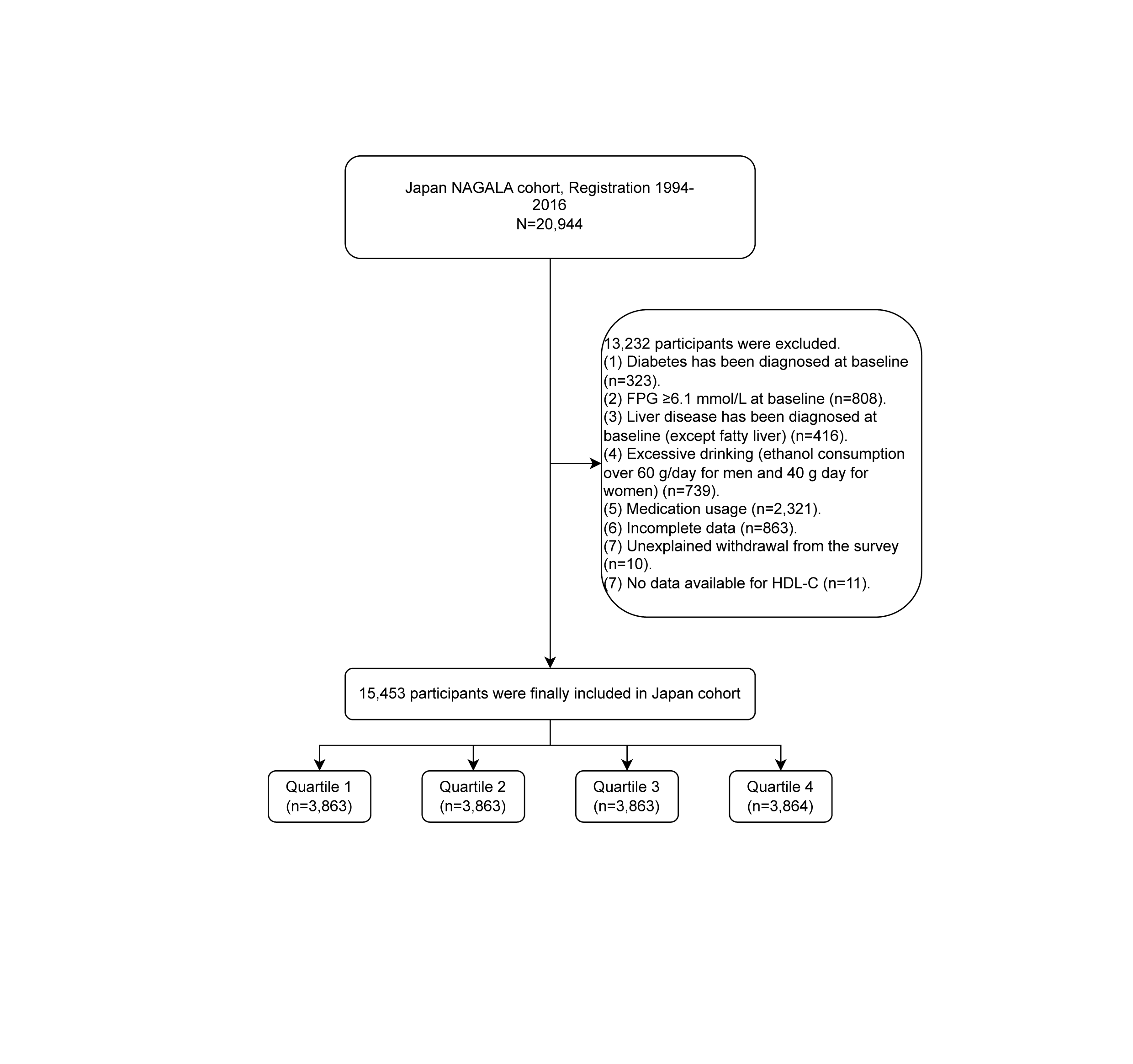


**Figure S1. Flowchart of the NAGALA cohort selection process.** From 20,944 initial participants (2004-2015), exclusions for diabetes, liver disease, alcohol overuse, and missing data yielded a final cohort of 15,453 individuals. Detailed exclusion criteria and attrition rates are shown.


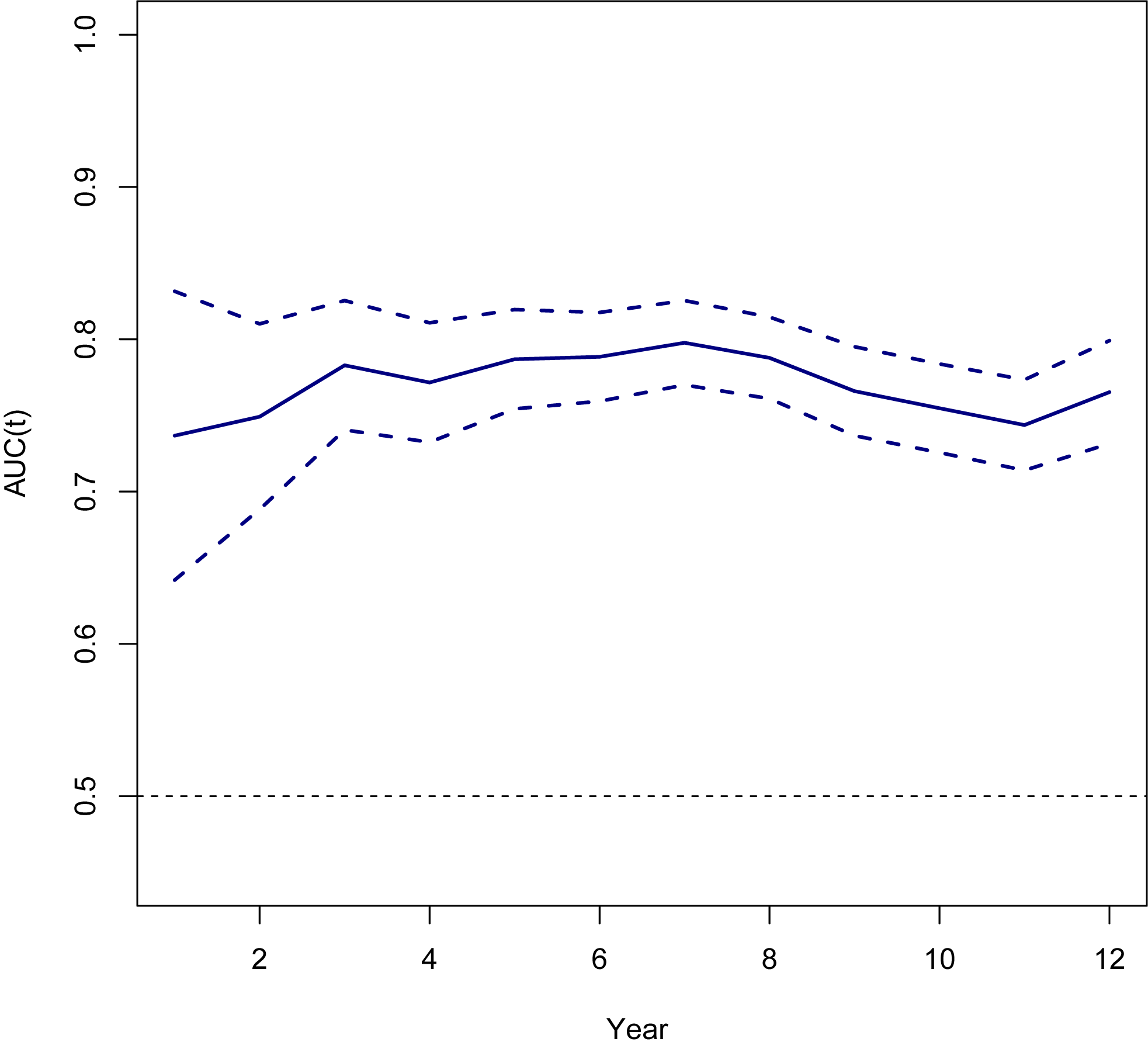


**Figure S2. Time-dependent ROC analysis of CHG’s predictive capacity for incident T2DM over 12 years.** Predictive accuracy peaked at 7 years (AUC = 0.798, 95% CI:0.77–0.825) and remained robust until 8 years (AUC = 0.788). Performance gradually declined thereafter but maintained clinical utility (AUC > 0.74) throughout the follow-up period.
